# Gender difference in handgrip strength of Italian children aged 9 to 10 years

**DOI:** 10.1186/s13052-016-0226-y

**Published:** 2016-02-19

**Authors:** Tiziana Montalcini, Yvelise Ferro, Maria Antonietta Salvati, Stefano Romeo, Roberto Miniero, Arturo Pujia

**Affiliations:** Nutrition Unit, Department of Experimental and Clinical Medicine, University Magna Grecia, Catanzaro, 88100 Italy; Nutrition Unit, Department of Medical and Surgical Science, University Magna Grecia, Catanzaro, 88100 Italy; Pediatric Unit, Department of Medical and Surgical Science, University Magna Grecia, Catanzaro, 88100 Italy; Metabolic Disease Unit, Department of Molecular and Clinical Medicine, University of Gothenburg, Gothenburg, 40530 Sweden

**Keywords:** Handgrip strength, Dynamometer, Nutrition, Children, Early phase of puberty

## Abstract

**Background:**

Handgrip strength is an index of nutritional status which correlates to morbidity and mortality in young. It differs in adults and adolescents between gender. However, it is no clear whether a difference exists also in children aged 9 to 10 years, and which are the factors influencing it. Furthermore, data of Handgrip strength of Italian schoolchildren are lacking. Aim of this study was to provide Handgrip strength value from a sample of healthy Italian schoolchildren aged 9 to 10 years and to determine which factors affect grip strength at this age.

**Methods:**

We enrolled 137 children (boys *n* =66; girls *n* = 71) who underwent a body mass index and Handgrip strength measurement. Handgrip strength was assessed by an hydraulic hand dynamometer.

**Results:**

The mean handgrip strength value was 13.8 ± 4.0 for girls and 15.2 ± 3.0 kg for boys (*p* = 0.04) thus, we found a significant difference between gender. We have not found a significant differences in anthropometric parameters between gender. In the univariate analysis Handgrip strength was associated with age, BMI, height, weight and gender (*p* < 0.001 for age, *p* < 0.001 for BMI, *p* < 0.001 for height, *p* < 0.001 for weight and *p* < 0.04 for gender). The Multivariate linear regression analysis showed that age, BMI and gender were all correlated to grip force.

**Conclusion:**

We found a significant difference in grip strength between healthy Italian schoolchildren aged 9 to 10 years. This parameter seems to be primarily influenced by gender. Our investigation is important since currently data regarding the reference values of HGS for Italian children are lacking.

## Background

Physical fitness has been identified as a predictor of morbidity [1–3] and mortality [4] in the young.

In particular, muscular fitness has been proven to be associated with insulin sensitivity in both children and adolescents [5] which, in turn, is linked to the future risk of type 2 diabetes mellitus (T2DM) in adulthood [6]. The role of a good muscular fitness has been also well recognised in the prevention of several chronic disease [7]. In this regard, Handgrip Strength (HGS) is an index of muscular fitness, also commonly used in both adults and youths as a marker of nutritional status [8]. In fact, an association between some micronutrient deficiencies, which are common in European young people [9], and HGS has been found [10]. Furthermore, this parameter correlates to several disease and clinical complications [11–14] and can predict mortality in both adults and youths [4, 15, 16].

Currently, there are different investigations proposing reference values for HGS [4, 17–20], and some of which have been performed exclusively in young Europeans [18–20]. Unfortunately, there is insufficient of data for European schoolchildren aged 9 to 10 years, especially Italian [18, 19]. Nevertheless the IDEFICS study was performed in European children, including those from Italy, HGS was provided only for girls and boys at age 9 [18]. The ELENA study examined HGS in a large cohort of young people from several European countries, but they were aged over 12 years [19]. Other investigations provided the HGS value of only Spanish [20, 21] or Estonian children [22] aged 9 to 10 years. Reference values are necessary to identify children with malnutrition or at risk of other clinical complications and to plan appropriate therapeutic interventions.

Furthermore, it has been suggested that several anthropometric and body composition variables could have a role in influencing HGS, but the most important is currently unclear. Some authors highlighted the influence of body height on HGS in the early stage of puberty, especially in boys. [23, 24] A parallel increase of HGS has been also demonstrated with age [18, 20], that seems largely dependent on the increase in body mass index (BMI) and, particularly, muscle mass [25]. However, this could simply reflect the sexual dimorphism [26], due to the action of sex steroid hormones.

Consequently, since there is no data on the HGS for Italian schoolchildren aged 9 to 10 years, in this investigation we sought to measure HGS values in apparently healthy Italian children aged 9 to 10 years. Furthermore, in this study we investigated which factors, also including anthropometric factors, affect HGS at this age.

## Methods

Two hundred Italian boys and girls volunteers from one primary public school were invited to participate in this observational study. Following agreement with the schools, anthropometric measurements and an HGS assessment were performed after parental consent obtained via letter which explained aims and procedures of the study. We planned to exclude those with clinical evidence of debilitating diseases and those practicing competitive sport.

Thus, parents were requested to inform the researchers of any clinical condition or illness affecting participants or, alternatively, to exclude their son from the study participation. A total of 63 children refused to participate in the study without explanations from their parents; We therefore enrolled 137 children (boys: *n* =66; girls: *n* = 71) who underwent an anthropometric assessment. The investigation conforms to the principles outlined in the Declaration of Helsinki. Written informed consent was obtained from each individual and patient’s legal surrogate. Since the study design (cross-sectional) it was not necessary to consult the local ethics committee.

### Anthropometric measurements

All tests were performed in a dedicated room after a 12 h overnight fasting. Body weight was measured with the subjects lightly dressed, subtracting the weight of clothes. Body weight was measured with a calibrated scale and height measured with a wall-mounted stadiometer and were recorded to the nearest 0.1 kg and 1 mm respectively. BMI was calculated with the following equation: weight (kg)/height (m)2.

### HGS measurement

The maximal HGS was measured by dieticians previously trained in the technique. The HGSwas measured using an hydraulic hand dynamometer (Hersteller/manufactures; SAEHAN Corporation, Masan-Korea; Distributor Rehaforum Medical GmbH, Elmshorn-Germany) having less than 10 % variation in results for various grip positions. Subjects were seated, with their elbows flexed at 90° and supported at the time of the measurement. Children was instructed to squeeze the dynamometer as strong as possible by each hand, for three consecutive times and the highest value (maximal value in kilograms), was used for the analyses [27].

### Statistical analysis

Data are reported as mean ± (SD). A *t*-test was performed to compare the means between gender.

The Pearson correlation was used to identify the variables correlated to the HGS given that the continuous variables were normally distributed according to the Kolmogorov-Smirnov test (age, weight, height, BMI). The Multivariate linear regression analysis was used to test the association between HGS and the variables selected among all that, in the univariate analysis, correlated with HGS with a *p* < 0.1. To better understand which factors were associated to HGS, in a first model we included BMI as independent variables, while in a second model we replace BMI with weight and height. Significant differences were assumed to be present at *p* < 0.05 (two-tailed). All comparisons were performed using SPSS 20.0 for Windows (IBM Corporation, New York, NY, United States).

## Results

The mean HGSvalue was 13.8 ± 4.0 for girls and 15.2 ± 3.0 kg for boys kg (*p* = 0.04). The characteristics of the population according to gender are shown in Table 1. None of them practiced activities affecting our assessment.

**Table 1:** Anthropometric characteristics and handgrip strength value of the subjects

Variables	Girls	Boys	*P*-values
	Mean ± SD	Mean ± SD	
	(*n* = 71)	(*n* = 66)	
Age (years)	9.4 ± 7	9.5 ± 7	0.19
Weight (kg)	39.2 ± 11	39.0 ± 9	0.91
Height (m )	1.39 ± 0.07	1.40 ± 0.07	0.37
BMI (Kg/m^2^)	19.9 ± 4	19.6 ± 3	0.65
HGS (kg)	13.8 ± 4	15.2 ± 3	0.043

*BMI* Body mass index, *HGS* Handgrip strength

**Fig. 1:**
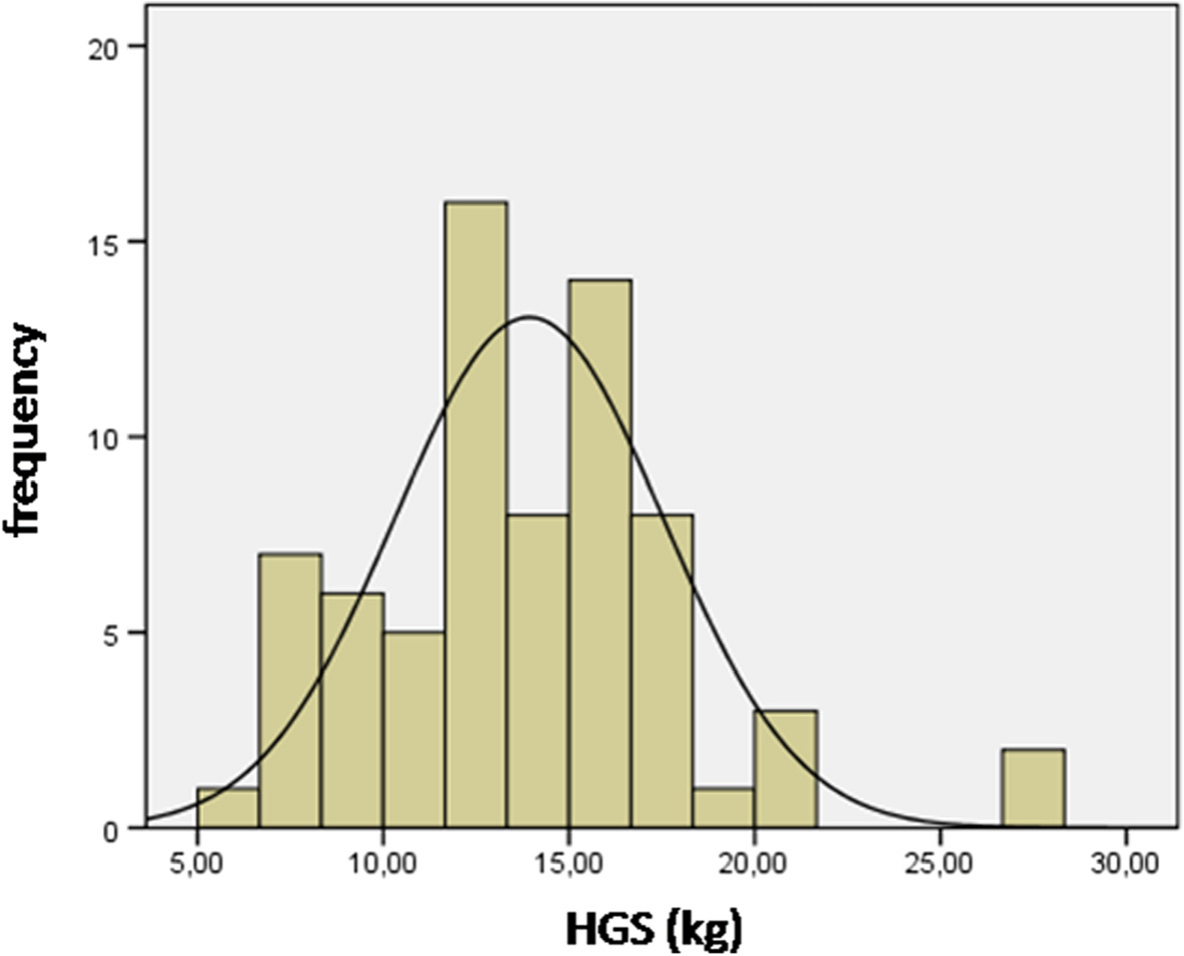
Normal distribution of HGS for girls. Legend- HGS, handgrip strength; Kg, kilogram

**Fig. 2:**
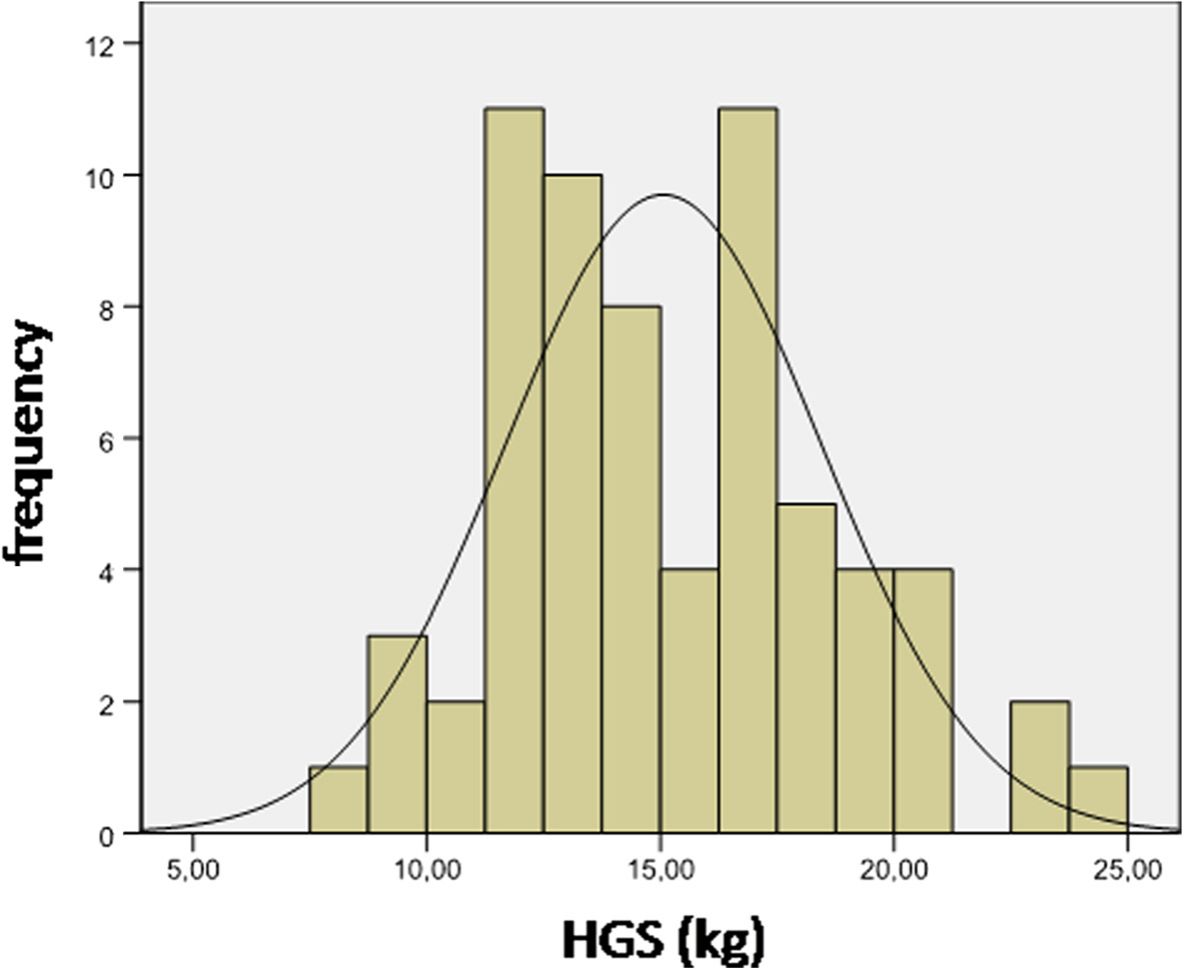
Normal distribution of HGS for boys. Legend- HGS, handgrip strength; Kg, kilogram

Figures 1 and 2 shown normal distribution of HGS for girls and boys, respectively.

Table 2 shows the factors associated with the HGS (*p* < 0.001 for age, *p* < 0.001 for BMI, *p* < 0.001 for height, *p* < 0.001 for weight and *p* < 0.04 for gender). The Multivariate linear regression analysis (Table 3) showed that age, BMI and gender were all correlated to HGS (Model I: *p* < 0.001 for age, *p* < 0.001 for BMI and *p* < 0.04 for gender). When we replace BMI with weight and height, the association with gender disappeared (model II: *p* < 0.001 for age, *p* < 0.001 for height, *p* < 0.001 and for weight).

**Table 2:** Pearson’s correlations-factors correlated with HGS

Variables		Age	Weight	Height	BMI	Gender
HGS	r	0.41	0.55	0.59	0.39	0.17
	p	<0.001	<0.001	<0.001	<0.001	0.04

*BMI* Body mass index, *HGS* Handgrip strength

**Table 3:** Multivariate linear regression analysis-factors associated with HGS

Dependent variable	B	SE	β	p	95 % CI		Tolerance	VIF
HGS					Upper limit	Lower limit		
Independent variables								
Model I								
Age	2.08	0.37	0.39	<0.001	1.34	2.82	0.98	1.01
BMI	0.35	0.06	0.39	<0.001	0.23	0.48	0.99	1.00
Gender	1.13	0.56	0.14	0.047	0.01	2.25	0.98	1.01
Model II								
Age	1.13	0.39	0.21	<0.001	0.36	1.90	0.76	1.30
Height	15.16	4.77	0.29	<0.001	5.73	24.6	0.49	2.02
Weight	0.12	0.03	0.33	<0.001	0.06	0.18	0.60	1.64

Exclude variables in Model II: gender

*BMI* Body mass index, *HGS* Handgrip strength, *VIF* Variance inflation factor

## Discussion

In this investigation we provided the maximum HGS value of apparently healthy Italian schoolchildren girls and boys aged 9 to 10 years. Furthermore, as expected, we demonstrated a significant difference in HGS performance between gender (Table 1). We also found an association between HGS and age, gender and anthropometric variables such as BMI, height and weight (Tables 2 and 3).

Our investigation is important since, currently, there is scarcity of data regarding the reference values for HGS in European children aged 9 to 10 years, especially Italian.

The availability of reference values assumes importance in a number of conditions. HGS is used as index of physical fitness in children [1–4]. Physical activity plays a key role in the normal growth during the first phase of life and for the psycho-social maturation of the young [28] and physical fitness is a predictor of morbidity [1–3]. HGS is a nutritional status index [8, 10, 29, 30] and is useful for investigating patients with neuromuscular disease [31]. Moreover, HGS is a predictor of mortality in youth [4]. Therefore, the availability of normative data for HGS could help to identify those children needing special dietary or pharmacological treatments or those at high risk of clinical complications [11–14].

In line with other investigations, like EUROFIT [20] and CHMS [17], we measured the maximal HGS, while in the IDEFICS [18] and HELENA [19] studies the authors used the mean value between hands. However, both methods were found to be highly consistent with no statistically significant difference [27]. Our HGS values were similar to those of EUROFIT [20] and IDEFICS [18]. On the contrary, as expected, our result was very different from those of CHMS, performed in Canadian children with an HGS value between 24 and 27 kg in boys and between 21 and 24 in girls aged 8–10 years old [17]. This finding reinforces the importance of having a reference value for different populations.

An important finding of our investigation was the significantly greater maximal HGS in boys than in girls. In this regard, we found, in the whole population, an HGS value of 13.8 ± 4.0 in girls and 15.2 ± 3.0 kg in boys (Table 1). Our results were similar to those of the EUROFIT study, in which the maximal HGS was 14.1 ± 2 in girls and 15.7 ± 3 kg in boys at age 9 and 17.1 ± 3 in girls and 16 ± 3 kg in boys at age 10 [20]. Even in the CHMS, Helena and IDEFICS studies, a sex-dependent difference was found [17–19]. Nevertheless it has been demonstrated that sex- and age- dependent differences in HGS disappears when HGS is normalised for fat-free mass [25], the most import factor influencing HGS seems to be still gender or, specifically, sex hormones. In fact, fat-free mass, in turn, is linked to the sex hormones and is more represented in boys than in girls [25]. In this regard, it is well known that sexual dimorphism in body composition is largely due to the action of sex steroid hormones [26]. An important concept is that sex difference in body composition is manifest from foetal life and, in children, a significant difference in estradiol and testosterone is evident before the external signs of puberty appear [32], probably leading to a difference in HGS. Interestingly, a specific sexually dimorphic response to maternal diet was observed in the placenta [33]. Placenta reacts differently to the same environment depending on the sex of the foetus [33, 34]. All together, these studies confirm the validity of our data and suggest a different response early in life between gender leading to a different body composition and HGS. According with this assumption, we did not find a significant difference in anthropometric parameters (height, body mass and BMI) between genders (Table 1).

Previous investigations demonstrated that HGS is positively correlated with weight, height and body surface area [23, 35] and that, in the early phase of puberty, body height could be a key factor influencing HGS [24]. Our results are in line with these previous findings. In fact, when we included in the multivariable analysis, as independent variable body height and weight, in place of BMI, the gender factor disappeared (Table 3). However, the height of children is considered an index of their sexual maturity [36]. Thus, again, the most import factor influencing HGS could be gender.

Also the age-dependent increase in HGS, previously described [18, 20] can be attributed to hormonal changes over time [37], nevertheless in boys, growth hormone and testosterone have more effects on HGS than in girls [37].

Our study highlights the need to have a reference value for HGS useful to plan interventions aimed at improving fitness in children, especially in those with a low HGS. Components of prenatal, prepubertal and pubertal growth have long-term consequences for midlife HGS [38]. Early interventions that facilitate building muscle mass could also prevent morbidity and mortality in later life [39–41].

Unfortunately, measuring HGS is not commonly practiced in pediatric population, despite the low cost and portability of the device, thus, our study suggests to implement the measure HGS.

We should mention some limitations of this study. First, our findings should be interpreted with caution, due to the place where they originated. However, this investigation was carried out on representative samples of the Italians children potentially increasing knowledge on this issue from a geographical perspective. In addition, we did not performed the nutrient intake assessment or used any questionnaires evaluating performance status in this sample and a medical examination was not carried out. We sought to facilitates the participation of the children. However, parents were requested to inform the researchers of any clinical condition affecting participants or, alternatively, to exclude their son from the study. It was possible that this fact affected data. However, a total of 63 children excluded themselves from the study, probably on the basis of our exclusion criteria. Nevertheless data on the physical activity are lacking, in this study we excluded those practicing competitive sport but included those practicing a recreational sport, since it could be assuming a relevant influence on HGS only in the first [42]. Of course, other unmeasured confounders could exist explaining the observed associations. Finally, our study is limited by its small size. However, the statistical analysis is adequate. Our results were not purely random as established by a previous investigation [20, 21] and were confirmed by multiple statistical analyses.

## Conclusion

In this investigation, we found a significant difference in HGS between apparently healthy Italian schoolchildren girls and boys aged 9 to 10 years. HGS seems to be primarily influenced by gender. Our investigation is important since currently data regarding the reference values for HGS in Italian children at this age are lacking, thus we provided reference values.
